# Editorial: Severe bleeding events among critically ill patients with hematological malignancies

**DOI:** 10.1186/s13613-024-01401-3

**Published:** 2024-11-08

**Authors:** Nathan D. Nielsen, Jean-Marc Tadié, Raphaël Clere-Jehl

**Affiliations:** 1 Division of Pulmonary, Critical Care and Sleep Medicine & Section of Transfusion Medicine and Therapeutic Pathology, University of New Mexico School of Medicine, Albuquerque, USA; 2 Infectious Diseases and Intensive Care Unit, Pontchaillou University Hospital, 2 rue Henri Le Guilloux, Rennes, 35033 France; 3 UMR 1236, Univ Rennes, INSERM, Etablissement Français du Sang Bretagne, Rennes, France; 4 Médecine Intensive et Réanimation, Hôpital de Hautepierre, University Hospital of Strasbourg, Strasbourg, France; 5 Laboratoire d’ImmunoRhumatologie Moléculaire, INSERM (French National Institute of Health and Medical Research), UMR_S1109, Centre de Recherche d’Immunologie et d’Hématologie, University of Strasbourg, Strasbourg, France

**Dear Editor,**

Severe bleeding is a life-threatening complication that is particularly feared in critically ill hematological patients. However, the rate of ICU-acquired major bleeding events in these patients is generally underinvestigated, with only small series on targeted hematological diseases previously available [1] or appearing in secondary outcomes [2]. As such, the original article “Severe bleeding events among critically ill patients with hematological malignancies” by Vigneron et al. provides very useful data on this topic.

In this interesting work, severe bleeding events, defined as grades 3 or 4 in the World Health Organization (WHO) classification, occurred in 10.8% of a total population of 1012 patients. The authors considered only ICU-acquired hemorrhage, i.e. bleeding events arising at least 24 h after ICU admission. In 28% of the patients the bleeding event was a recurrence from a prior bleeding episode, demonstrating that the other 72% of cases were not admitted to the ICU for this reason (new-onset bleeding), thus raising questions about risk prediction.

Even more than the significant (10.8%) rate of ICU-acquired bleeding events, however, it is the severity of the bleeding events that underscores their clinical importance. Indeed, 57.8% of severe bleeding events were grade 4, *i.e*.: potentially life-threatening hemorrhage. Moreover, patients with bleeding events experienced significantly higher ICU mortality (55% vs. 18% in non-bleeding patients), longer ICU stays (18 vs. 9 days) with more frequent need for supportive treatments (mechanical ventilation, vasopressors, renal replacement therapy), in addition to higher transfusion requirements. Of note, patients with ICU-acquired bleeding events displayed more extra-hematological organ failures at ICU admission, evidenced by higher SAPS II and non-platelet SOFA scores. This also suggests that overall patient severity is associated with a higher bleeding risk and that the need for organ support in the ICU is unlikely to be entirely the result of hemorrhagic sequelae.

Although the study is retrospective and does not include the administration of effective prophylactic therapies in mechanically ventilated patients such as proton-pump inhibitors [3], the study emphasizes the critical importance of early identification of patients at high risk for hemorrhagic complications. Identifying high-risk patients presents a complex diagnostic challenge because the degree of thrombocytopenia was not associated with severe bleeding and as such, does not reliably predict the risk of hemorrhage, in line with previous studies [1, 4]. However, refractoriness to platelet transfusion was associated with an increased risk of ICU-acquired bleeding, suggesting that platelet consumption or alloimmunization may play a role. However, there are no routinely available assays for assessing platelet consumption and alloimmunization testing can be complex and prolonged, making the assessment of platelet refractoriness difficult to establish within a clinically relevant time-frame.

Intuitively, a robust predictor of bleeding risk in these patients would be thought to be disseminated intravascular coagulation (DIC). However, the most commonly used DIC scoring systems, such as the International Society on Thrombosis and Haemostasis (ISTH) score, lead to the overdiagnosis of DIC in this population, mainly because thrombocytopenia of a central origin is extremely common due either to chemotherapy effects or to bone marrow invasion [4]. Other diagnostic scores have been proposed to define DIC in this population but many of them are either highly complex or rely upon non-widely accessible laboratory tests which prevents their common use. A notable exception is the DIC diagnostic score of the Japanese Ministry of Health and Welfare (JMHW), a platelet-free score which, while easy to apply to hematological patients, has not as yet been sufficiently evaluated for the prediction of bleeding events in this population [5].

This study also provides insights into the assessment of coagulopathy in these patients, in particular, as to which hemostatic parameters must be taken into consideration when gauging bleeding risk. It reinforces the impact of a prolonged prothrombin time (or elevated INR) on the risk of bleeding, a finding previously reported in critically ill hematological patients [1, 4]. This remains the only coagulation parameter observed to be consistently associated with severe bleeding in this population, whereas viscoelastic tests (TEG/ROTEM) lead to results that are difficult to interpret and as such are not currently recommended by the ISTH. Moreover, coagulopathy and DIC may lead to opposite consequences, because some patients experience a hyperfibrinolytic state, commonly associated with bleeding, whereas others develop a hypofibrinolytic state associated with thrombosis [6]. And while bleeding disorders are more frequent in thrombocytopenic patients, some hematological patients are at high risk of thrombotic events [2]. We currently still lack reliable biomarkers or scoring systems to accurately assess both thrombotic and bleeding risks in critically ill patients suffering from hematological malignancies. The treatment of hemostatic disorders in this population therefore remains a double-edged sword [6].

In sum, while the management of critically ill hematological patients has improved considerably over the last few decades, there is still a great deal that is unknown about the hemostatic imbalances in this population. While we applaud the important contribution of Vigneron and colleagues to this dialogue, there is clearly much further investigation required before we can collectively implement effective preventative strategies for this high-risk group of individuals.

## Data Availability

Not applicable.
